# Left ventricle morphological and functional characteristics associated to the presence and magnitude of myocardial fibrosis by CMR in hypertrophy cardiomiopathy

**DOI:** 10.1186/1532-429X-16-S1-P315

**Published:** 2014-01-16

**Authors:** Hélder Andrade Gomes, Mariana Lamacié, Fábio V Fernandes, Bernardo N Abreu, Juliana Bello, Carlos E Prazeres, Tiago A Magalhães, Adriano Carneiro, Valéria Moreira, Carlos E Rochitte

**Affiliations:** 1 Hospital do Coração - Associação do Sanatório Sírio, São Paulo, Brazil; 2 Spanish Society of Cardiology, Madrid, Spain; 3 Heart Institute, InCor, University of São Paulo Medical School, São Paulo, Brazil

## Background

Hypertrophic cardiomyopathy (HCM) is a common inherited cardiovascular disease present in one in 500 of the general population, and it is main cause of sudden death in young people. The presence of myocardial fibrosis in HCM is very common (more than 2/3 of patients), and its identification by late gadolinium enhancement (LGE) on cardiac magnetic resonance (CMR) helps the diagnosis and it is associated with ventricular arrhythmias and worse prognosis. The aim of our study was to find CMR characteristics that are related to the presence and burden of myocardial fibrosis by CMR in HCM.

## Methods

We evaluated 115 consecutive HCM patients who underwent cardiac MRI and analyzed both the presence/absence of myocardial fibrosis (LGE) and also its magnitude (estimated as absolute mass of myocardial fibrosis in grams and normalized to LV mass as percentage of LV myocardial mass) with other morphological and functional factors as well as population characteristics. Patients with a history of acute myocardial infarction as well as other cardiomyopathies that could present late enhancement were excluded.

## Results

The mean age was 46.6 ± 16.1 yo, 77% male. Eighty patients (70%) had myocardial fibrosis in cardiac MRI. Patients with myocardial fibrosis had lower ejection fraction of the left ventricle (68.8 vs. 74.6 ± 0.1%, p = 0.011), higher end-systolic volume (45 ± 24 [23 ± 11] vs. 33 ± 12 [18 ± 5] ml [ml/m2], p = 0.027 [p = 0.041]), greater maximum thickness of the ventricular wall (21.9 ± 5.2 vs. 16.4 ± 2.8 mm, p < 0.001) and increased left ventricular mass (192.8 ± 58.1 [98.5 ± 27.0] vs. 157.5 ± 50.4 [82.8 ± 21.5] g[g/m2], p < 0.001 [p = 0.001]). In a logistic regression analysis, only ejection fraction (p = 0.034) and maximum wall thickness (p < 0.001) were independently associated with the presence of fibrosis. Among those with myocardial fibrosis, a greater mass of fibrosis correlated with a lower ejection fraction (r = -0.42, p = 0.003), lower end-systolic volumes (r = 0.56, p < 0.001), greater maximum thickness wall (r = 0.28, p = 0.024) and increased left ventricular mass (r = 0.35, p = 0.017). On the multivariate analysis, only the stroke volume (p < 0.001) and maximum thickness (p = 0.020) were correlated with the mass of myocardial fibrosis. However, when we analyzed the burden of fibrosis in relation to left ventricular mass (percentage of fibrosis) only greater end-systolic volume was independently associated with a higher percentage of fibrosis (r = 0.48, p < 0.001).

## Conclusions

The presence of myocardial fibrosis in patients with HCM is independently related to lower ejection fraction and higher maximum thickness of the ventricular wall. The burden of fibrosis correlated independently with a higher maximum wall thickness (mass of fibrosis) and lower end-systolic volume (mass and percentage of fibrosis).

## Funding

No funding.

